# Intra-Amnionic Threonine Administered to Chicken Embryos Reduces *Salmonella* Enteritidis Cecal Counts and Improves Posthatch Intestinal Development

**DOI:** 10.1155/2018/9795829

**Published:** 2018-11-26

**Authors:** Alexandre L. de B. Moreira Filho, Celso J. B. Oliveira, Oliveiro C. Freitas Neto, Candice M. C. G. de Leon, Mauro M. S. Saraiva, Maria F. S. Andrade, Bryan White, Patrícia E. N. Givisiez

**Affiliations:** ^1^Departmento de Zootecnia, Centro de Ciências Agrárias, Universidade Federal da Paraíba (UFPB), Areia, PB 58397-000, Brazil; ^2^Departamento de Ciências Veterinárias, Centro de Ciências Agrárias, Universidade Federal da Paraíba (UFPB), Areia, PB 58397-000, Brazil; ^3^Department of Animal Sciences, Division of Nutrition Sciences, University of Illinois at Urbana-Champaign (UIUC), Urbana, IL 61801, USA

## Abstract

This study assessed the effect of *in ovo* threonine supplementation on the response of broiler chicks challenged with *Salmonella* Enteritidis, considering bacterial counts in cecal contents, intestinal morphology, body weight, and weight gain. Fertilized eggs were inoculated in the amniotic fluid with saline (NT) or 3.5% threonine (T) solution at day 17.5 of incubation. At hatch, chicks were individually weighed and cloacal swabs were screened for *Salmonella*. At 2 days of age, half of the birds from each *in ovo* treatment were given either 0.5 mL of nutrient broth (sham-inoculated) or nalidixic acid-resistant *Salmonella* Enteritidis (SE Nal^R^) in nutrient broth (8.3 × 10^7^ colony forming units (CFU) SE Nal^R^/mL). The birds were distributed using a completely randomized design with four treatments after the *Salmonella* challenge: no *in ovo* Thr supplementation and sham-inoculated in the posthatch challenge (NT-SHAM), *in ovo* Thr supplementation and sham-inoculated (T-SHAM), no *in ovo* Thr supplementation and SE Nal^R^-challenged (NT-SE), and *in ovo* Thr supplementation and SE Nal^R^-challenged (T-SE). *In ovo* threonine supplementation reduced *Salmonella* Enteritidis colonization 168-hour postinoculation and reduced the negative effects associated with *Salmonella* infection on intestinal morphology and performance, with results similar to those of the sham-inoculated birds. *In ovo* Thr supplementation increased the expression of *MUC2* at hatch and the expression of *MUC2* and *IgA* at 2 days of age and 168-hour postinoculation. Our results suggest that providing *in ovo* threonine promotes intestinal health in broilers challenged with *Salmonella* Enteritidis in the first days of life.

## 1. Introduction

Important transformations occur posthatch that are essential for chick survival and determinant for the broiler. The most important of these transformations is the rapid development of the gastrointestinal tract, which is necessary for enabling nutrient assimilation. During this stage, broilers are exposed to several types of microorganisms, but their defense mechanisms are minimally developed and are limited to the innate immune system and maternal antibodies.

The hatchling's gastrointestinal tract contains a small number of microorganisms [1], which facilitate pathogen colonization [2]. Susceptibility to *Salmonella* infections depends on age; younger animals up to 3 days old are more vulnerable to intestinal infections and more prone to developing inflammatory reactions and intestinal lesions than adult birds [3].

The mucosal layer is the first line of defense against bacteria and other pathogens [4]. Mucin is the main component of the protective mucosal layer, which protects the intestinal epithelium and prevents damage and infections by pathogenic bacteria. Mucin production is closely related to dietary threonine levels [5].

Evidence suggests that threonine (Thr) supplied to broilers in early life stages, e.g., during embryogenesis, may improve their immune response to *Salmonella* during posthatch. For example, *in ovo* Thr supplementation increased mucin (MUC2) expression in broiler chickens [6] and quails [7], thus increasing protection of the intestinal mucosa against pathogens and improving digestion and nutrient absorption.

Studies have shown that *in ovo* nutrition benefits the intestinal development [8, 9], increases the intestinal expression of glucose and amino acid transporters [9, 10], increases mucin synthesis in the intestinal epithelium [6, 7], improves the humoral immune response [11], and increases interleukin expression [6]. The present study evaluated the effect of *in ovo* Thr supplementation on the response of two-day-old broilers challenged with *Salmonella* Enteritidis.

## 2. Materials and Methods

All management, slaughter, and sampling procedures were approved by the Ethical Committee of Animal Use in Research of the Federal University of Paraíba (Comissão de Ética no Uso de Animais da Universidade Federal da Paraíba) under the protocol number CEUA 078/2017. The study was conducted in the Departamento de Zootecnia, Centro de Ciências Agrárias da Universidade Federal da Paraíba, Brazil.

### 2.1. Incubation and *In Ovo* Supplementation

Three hundred fertile eggs were obtained from a commercial hatchery (Guaraves Alimentos Ltda., Guarabira, PB, Brazil), with a mean weight of 67.0 ± 1.56 g. The eggs originated from Cobb500 breeders aged 44 weeks and were distributed into three artificial incubators (IP130, Premium Ecológica Ltda., Belo Horizonte, MG, Brazil). All incubators were maintained under standard incubation conditions from one to seventeen days (D17) at 37.7°C and 60% relative humidity, with automatic turning every two hours. On the 11^th^ day of incubation (D11), nonfertile eggs were discarded after candling.

The eggs were randomly distributed into two treatments (*n* = 150 per group) based on the Thr concentration to be injected *in ovo* (NT: 0.0% Thr; T: 3.5% Thr) on D17.5 according to Uni and Ferket [12]. Briefly, all eggs were cleaned with 70% ethanol and punctured at the air chamber end. Nutritive solution (1.0 mL) was warmed to 30°C and injected through the puncture in the amniotic fluid using 1 mL syringes and 21 G needles. Inoculated eggs were placed in hatching trays and incubated at 36.7°C. After hatching, chicks were weighed individually and incubation was assessed by chick weight at hatching, total hatchability, fertile hatchability, pipped eggs, and embryo mortality.

### 2.2. Treatments and Management

At hatch, chicks were identified with leg bands and individually weighed and cloacal swabs were sampled from 20 birds per incubator to screen for *Salmonella*. The birds were then distributed into a completely randomized experimental design with 4 treatments of 36 birds (repetitions) per treatment kept in boxes. Each box was equipped with a feeder and a drinker and covered with nylon mesh to avoid contamination between boxes by vectors such as flies.

A corn-soybean meal diet was formulated for the initial phase based on Rostagno et al. [13]. Levels were 22.20% CP, 2950 kcal/kg ME, 1.31% digestible lysine, 0.94% digestible methionine + cystine, and 0.852% digestible threonine. Birds were weighed individually at the beginning and end of the experimental period. Initial and final body weights were used to calculate weight gain.

### 2.3. Inoculation

The birds were challenged with *Salmonella* at two days old. Inoculum was prepared using one colony of nalidixic acid-resistant *Salmonella* Enteritidis (SE Nal^R^) in nutrient broth (Acumedia, USA) containing nalidixic acid (100 *μ*g/mL) for 24 h at 37°C, and then, an aliquot (0.1 mL) was cultured again for four hours at 37°C. Inoculum concentration was determined by plating a serial dilution (10^−1^ to 10^−6^) on brilliant green agar plates (Acumedia) with nalidixic acid (100 *μ*g/mL) and incubating at 37°C. *Salmonella* colonies were counted after 24 hours. All birds in each box were inoculated with 0.5 mL of *Salmonella* Enteritidis Nal^R^ (8.3 × 10^7^ CFU/mL) into the crop, except the sham-inoculated control birds, who received only nutrient broth.

The birds were distributed in a completely randomized design with four treatments after being challenged with *Salmonella*: no *in ovo* Thr supplementation and sham-inoculated in the posthatch challenge (NT-SHAM), *in ovo* Thr supplementation and sham-inoculated (T-SHAM), no *in ovo* Thr supplementation and SE Nal^R^-challenged (NT-SE), and *in ovo* Thr supplementation and SE Nal^R^-challenged (T-SE).

### 2.4. Microbial Analysis

At 24-, 96-, and 168-hour postinoculation (hpi) with *Salmonella* or at 3, 6, and 9 days of age, six birds per treatment were killed by cervical dislocation. Cecal contents were collected from all birds in each treatment (n = 6) and weighed before being serially diluted (10^−1^ to 10^−6^) with peptone water (Acumedia). Twenty microliter aliquots was cultured on brilliant green agar with nalidixic acid (100 *μ*g/mL) and incubated at 37°C for 24 hours. Colony counts were performed, and values were expressed in colony-forming units per gram of cecal content (CFU/g).

### 2.5. Histology Analysis

Four birds per treatment were killed, and the medial regions of the duodenum, jejunum, and ileum were sampled for histological analyses. Samples were rinsed with sterile saline, immersed in buffered formalin for 24 hours, and rinsed in 70% ethanol. Slides were mounted with 5 *μ*m sections and stained with hematoxylin and eosin. Two slides with five to seven sections were mounted per bird. Photomicrographs of the duodenal mucosa were taken using a digital camera with 12.1 megapixels (Sony Inc.) connected to a light microscope, with 1.7 optical zoom and 10x objective. The images were analyzed using ImageJ software [14]. Villus height (VH), crypt depth (CD), villus width at the apex (AW), and base width (BW) were measured. Ten measurements were made per bird, with a total of 60 measurements per variable per treatment. The mean value was calculated per variable per bird. The villus : crypt ratio was calculated (VH/CD), and villus area was calculated according to Sakamoto et al. [15]: (2*π*)(AW/2)(VH).

### 2.6. MUC2 and IgA mRNA Expression

Total RNA was isolated from four ileum samples per treatment (500 *μ*g) using the RNeasy® Mini Kit (Qiagen, Valencia, CA, USA) following the manufacturer's guidelines, and the quality and purity were assessed using NanoDrop 2000 (Thermo Scientific, Wilmington, DE, USA) based on 260/280 and 260/230 ratios. Reverse transcription was performed using an AffinityScript QPCR cDNA Synthesis Kit (Agilent Technologies, Santa Clara, CA, USA) according to the manufacturer's guidelines.

Relative quantification by real-time polymerase chain reaction (PCR) was performed using the Brilliant III Ultra-Fast SYBR QPCR Master Mix (Agilent Technologies). Cycling was performed using a Stratagene Mx3005P (Agilent Technologies). MUC2 primer sequences were 5′-ATGTTTTTGCATCCCATTGC-3′ (forward) and 5′-TGCGGTGGATTGTCAGAATA-3′ (reverse); IgA primer sequences were 5′-ACCACGGCTCTGACGTACAC-3′ (forward) and 5′-CGATCGTCTCCTTCACAGCA-3′ (reverse), and *β*-actin primer sequences were 5′-ACCACTGGCATTGTCATGGACTCT-3′ (forward) and 5′-TCCTTGATGTCACGGACGATTTCC-3′ (reverse). Primers were designed using Primer Express (version 3, Applied Biosystems, Foster City, CA, USA).

Relative MUC2 and IgA mRNA abundance was determined using the 2^−ΔΔCt^ method [16]; Ct values for each sample were standardized for *β*-actin RNA.

### 2.7. Statistical Analysis

Microbial data were analyzed using a completely randomized design with two treatments, considering only treatments T-SE and NT-SE, with six replicates per treatment and sampling age (24, 96, and 168 hpi). Morphometry, gene expression, and performance data were analyzed using a completely randomized design with two treatments for samplings at posthatch and two days of age and four treatments for samplings at 24, 96, and 168 hours after inoculation with *Salmonella*. When significant differences were found, means were compared using Tukey's test and *F* test at *p* ≤ 0.05. Analyses were calculated using SAS [17].

## 3. Results

*In ovo* Thr supplementation did not affect hatchability (83% vs. 80%) or embryo mortality (17% vs. 20%). *In ovo* supplementation with 35 mg of Thr resulted in higher weight (*p* ≤ 0.05) at hatch when compared to birds not supplemented *in ovo* (53.36 g vs. 51.19 g, respectively).

**Figure 1:**
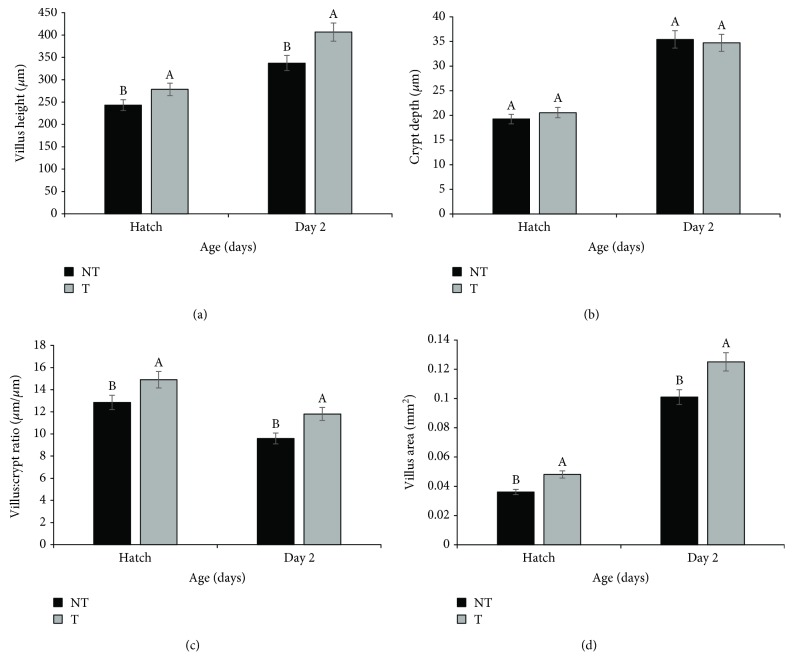
Villus height (a), crypt depth (b), villus : crypt ratio (c), and villus area (d) in the ileum at hatch and at two days of age in broilers supplemented with Thr *in ovo* (NT: 0.0% Thr; T: 3.5% Thr). The error bars represent the standard deviation.

Villus height, crypt depth, villus : crypt ratio, and villus area in the ileum showed similar results at hatch and at two days of age (Figure 1). All studied parameters were higher for animals supplemented with Thr *in ovo* (T) (*p* ≤ 0.05), but crypt depth was not different (*p* > 0.05). This was similar in the duodenum and jejunum, except for villus height at hatch and villus area at both ages in the jejunum, which did not significantly differ between treatments (*p* > 0.05) (Supplementary Table 1).

**Figure 2:**
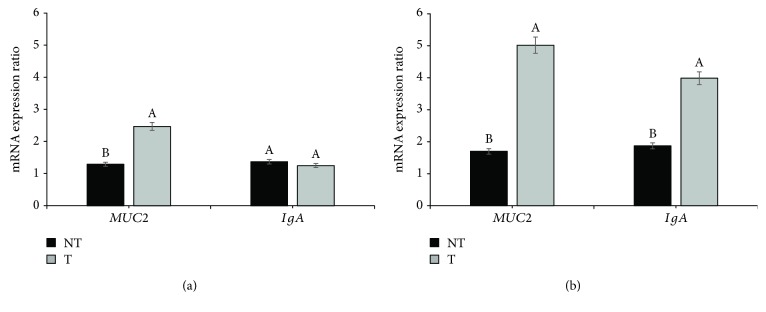
Relative expression of mucin (*MUC2*) and immunoglobulin A (*IgA*) mRNA at hatch (a) and two days of age (b) in broilers supplemented with Thr *in ovo* (NT: 0.0% Thr; T: 3.5% Thr). The error bars represent the standard deviation.

*In ovo* Thr supplementation affected the relative expressions of *MUC2* and *IgA* at hatch and two days of age (Figure 2). Thr supplementation resulted in increased expression of *MUC2* at hatch (*p* ≤ 0.05), but not of *IgA*, which did not significantly differ from the control group (*p* > 0.05). At two days of age, *MUC2* and *IgA* expressions were higher with Thr supplementation than for the control group (*p* ≤ 0.05).

**Figure 3:**
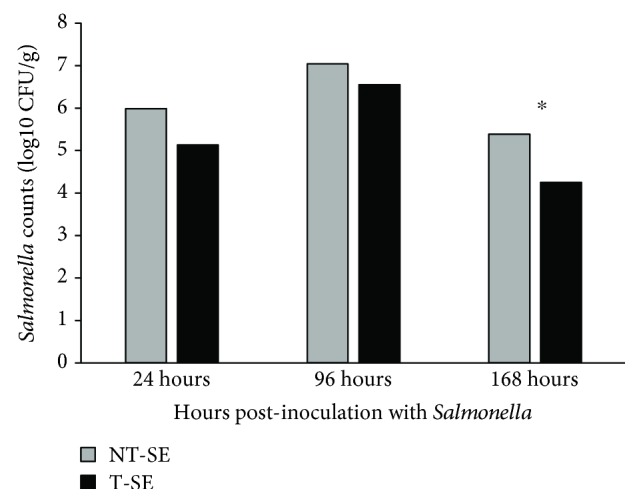
Bacterial counts in cecal contents (CFU/g) in broilers challenged with *Salmonella* Enteritidis (SE) and supplemented with Thr *in ovo*. NT-SE = no *in ovo* Thr supplementation and SE Nal^R^-challenged; T-SE = *in ovo* Thr supplementation and SE-challenged.

*In ovo* Thr supplementation had no effect (*p* > 0.05) on *Salmonella* cecal counts at 24 and 96-hour postchallenge; however, *Salmonella* cecal counts were lower with Thr supplementation at 168-hour postchallenge (*p* ≤ 0.05) (Figure 3). *Salmonella* was not recovered from sham-inoculated control birds.

**Figure 4:**
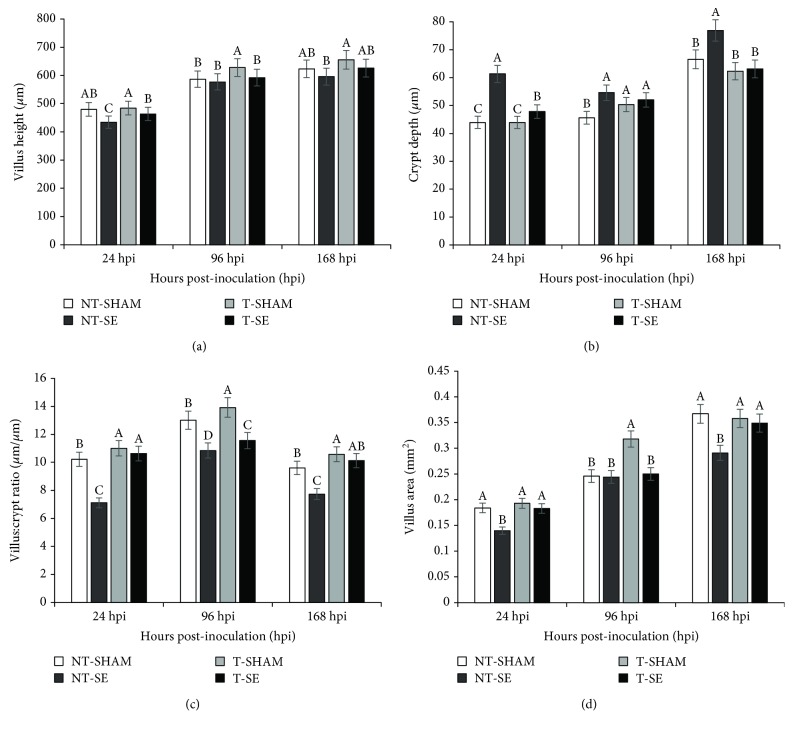
Villus height (a), crypt depth (b), villus : crypt ratio (c), and villus area (d) in the ileum in broilers at 24, 96, and 168-hour postinoculation (hpi) with *Salmonella*. NT-SHAM: no *in ovo* Thr supplementation and sham-inoculated in the posthatch challenge; T-SHAM: *in ovo* Thr supplementation and sham-inoculated; NT-SE: no *in ovo* Thr supplementation and SE Nal^R^-challenged; and T-SE: *in ovo* Thr supplementation and SE Nal^R^-challenged. The error bars represent the standard deviation.

The changes in villus height, crypt depth, villus : crypt ratio, and villus area in the ileum at 24, 96, and 168 hpi were similar (Figure 4). Villus height, villus : crypt ratio, and villus area were higher for T-SHAM, and crypt depth was higher (*p* ≤ 0.05) for NT-SE at 24, 96, and 168 hpi, but crypt depth at 96 hpi was not different between T-SHAM, T-SE, and NT-SHAM (*p* > 0.05). The T-SE group presented a higher or similar villus height and area than NT-SHAM in the three periods (Figure 4).

Similar results were observed in the duodenum and jejunum at 24 hpi (Supplementary Table 2). Villus height, villus : crypt ratio, and villus area were higher for T-SHAM (*p* ≤ 0.05), and the groups NT-SHAM and T-SE showed similar results. At 96 hpi, duodenal and jejunal villus height, villus : crypt ratio, and villus area were higher for broilers from unchallenged treatments (NT-SHAM and T-SHAM) (*p* ≤ 0.05) (Supplementary Table 3). Interestingly, the group supplemented with Thr *in ovo* and challenged with *Salmonella* (T-SE) presented similar villus heights and areas to the unchallenged groups. Crypt depth for both intestinal segments was higher for the treatment challenged with *Salmonella* and not supplemented with Thr *in ovo* (*p* ≤ 0.05).

At 168 hpi (Supplementary Table 4), villus height, villus : crypt ratio, and villus area in the duodenum and jejunum were higher in the T-SHAM group (*p* ≤ 0.05). Duodenal and jejunal villus height and villus area were similar between T-SHAM, NT-SHAM, and T-SE. The NT-SE group presented the highest crypt depth and lowest villus area and villus : crypt ratio (*p* ≤ 0.05). The lowest crypt depths were seen in T-SHAM and T-SE birds.

**Figure 5:**
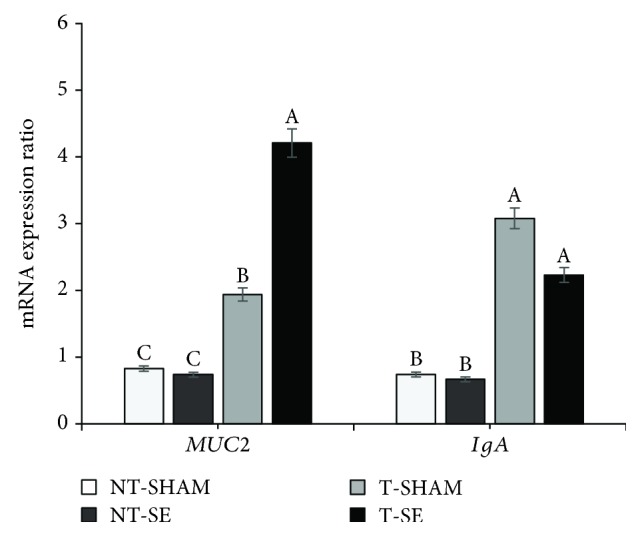
Relative expression of mucin (*MUC2*) and immunoglobulin A (*IgA*) mRNA in broilers at 168-hour (9 days of age) postinoculation with *Salmonella*. NT-SHAM: no *in ovo* Thr supplementation and sham-inoculated in the posthatch challenge; T-SHAM: *in ovo* Thr supplementation and sham-inoculated; NT-SE: no *in ovo* Thr supplementation and SE Nal^R^-challenged; and T-SE: *in ovo* Thr supplementation and SE Nal^R^-challenged. The error bars represent the standard deviation.

Relative expression of mucin (*MUC2*) in the ileum at 168 hpi was highest for T-SE (*p* < 0.01), followed by T-SHAM. Relative expression of immunoglobulin A (*IgA*) was also higher for T-SHAM and T-SE (*p* ≤ 0.05). For both genes, expression was lower for NT-SHAM and NT-SE (Figure 5).

**Figure 6:**
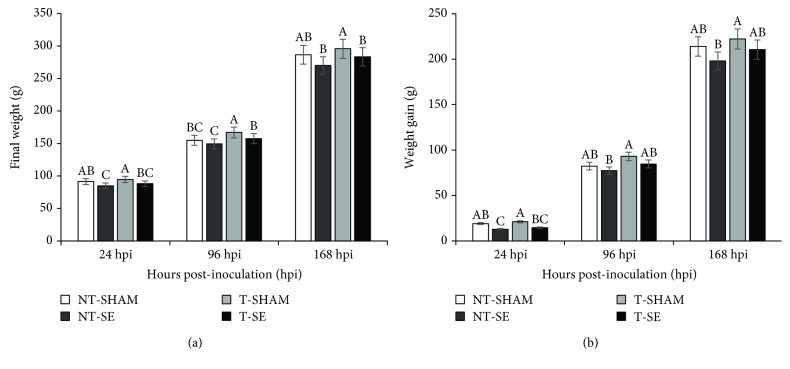
Final weight (3, 6, and 9 d) and weight gain in broilers supplemented *in ovo* with Thr and challenged with *Salmonella* Enteritidis posthatch at 24, 96, and 168-hour postinoculation (hpi). NT-SHAM: no *in ovo* Thr supplementation and sham-inoculated in the posthatch challenge; T-SHAM: *in ovo* Thr supplementation and sham-inoculated; NT-SE: no *in ovo* Thr supplementation and SE Nal^R^-challenged; and T-SE: *in ovo* Thr supplementation and SE Nal^R^-challenged. The error bars represent the standard deviation.

At 24 hpi, no significant differences in initial weight were observed between the four treatments (*p* > 0.05). Final weight and weight gain (Figure 6) were higher for T-SHAM (*p* ≤ 0.05) and not different from NT-SHAM. NT-SE presented the lowest final weight and weight gain (*p* ≤ 0.05).

Similar results were observed at 96 and 168 hpi (Figure 6); mean final weight and weight gain were highest for T-SHAM (*p* ≤ 0.05). However, at 168 hpi, T-SHAM was not different from NT-SHAM. Similar to what was observed at 24 hpi, NT-SE presented the lowest final weight and weight gain at 96 and 168-hour postinoculation. T-SE presented a similar final weight and weight gain to NT-SHAM in all sampling times (*p* > 0.05).

## 4. Discussion

The present results show that *in ovo* Thr supplementation increased *MUC2* and *IgA* expressions, positively affected histological and performance parameters, and decreased *Salmonella* Enteritidis cecal counts (168 hpi). Furthermore, *in ovo* Thr supplementation did not affect hatchability and supplemented chicks weighed more at hatch.

The benefit of *in ovo* supplementation on live weight at hatch may be related to the use of energy reserves during embryogenesis. Gluconeogenesis from protein catabolism may be only source of glucose for the embryo at hatch, thus negatively affecting broiler growth and development and likely leading to decreased body weight [8]. In the present study, this negative effect may have been alleviated by *in ovo* Thr supplementation because Thr is a gluconeogenesis substrate. Therefore, *in ovo* Thr supplementation may have increased the embryo's energy status by decreasing muscle protein depletion, thereby increasing body weight at hatch.

Similarly, higher body weight at hatch was seen after supplementing 30 mg and 35 mg Thr *in ovo* with only a small decrease in hatchability when different levels of Thr supplementation were used [18]. No negative effects on hatchability and beneficial effects on hatchling weight were observed when Thr [11, 19] or other amino acids [20, 21] were supplemented *in ovo*. Several factors related to the method of *in ovo* nutrient injection, including volume, temperature, concentration, site, embryo age, and egg handling, may affect the hatch rate and other parameters.

Our results also showed that *in ovo* Thr supplementation benefitted gastrointestinal tract (GIT) development. Thr-supplemented animals presented higher villus height, villus : crypt ratio, villus area, and *MUC2* and *IgA* expressions on the day of hatch and at two days of age. Previous studies have suggested improved intestinal development in response to *in ovo* nutrient supplementation, possibly related to the nutrients stimulating the intestinal mucosa at an earlier stage [9, 22, 23]. *In ovo* supplementation guarantees availability of nutrients and cofactors necessary to sustain and even accelerate enteric development, which may otherwise be limited by low nutrient availability at the end of incubation [11].

Kadam et al. [11] tested different levels of *in ovo* Thr supplementation and observed higher feed intake in broilers supplemented with Thr. This may be related to better functional development of the GIT with greater density of intestinal goblet cells in the intestines of animals supplemented with Thr, because Thr is an essential component of enzymes and mucin [11]. Tahmasebi and Toghyani [19] observed longer jejunum and ileum in animals supplemented *in ovo* with Thr, arginine (Arg), and Thr + Arg than in nonsupplemented animals. Regarding intestinal morphology parameters, the authors observed increased ileum villus height in animals supplemented with Thr and Arg.

Dietary supplementation with both Thr and mannan oligosaccharide (MOS) [5] or only Thr [24] has been reported to decrease the number of *Salmonella*-positive animals and bacterial counts in the cecal contents, similar to the present finding with *in ovo* Thr supplementation. Some factors may have contributed to decreased *Salmonella* cecal counts in the Thr-administered group at 168-hour postinoculation (9 days of age). The first is the development of the gut-associated lymphoid tissue (GALT). GALT remains immature up to three weeks posthatch, and intestinal protection is conferred by innate immunity and maternal antibodies, which may prevent or control pathogen translocation or intestinal spread [25].

Another important factor is the establishment of the commensal microbiota that becomes similar to that of the adult chickens around the second-week posthatch. Commensal intestinal bacteria protect the host from pathogen colonization by competing with the pathogens for epithelial binding sites and available nutrients and by producing bacteriocins, strengthening the intestinal immune response [26]. Commensal intestinal bacteria can also inhibit pathogenic bacteria from adhering to the intestinal mucosa by increasing intestinal mucin production [4]. In addition, Faure et al. [27] showed that some specific amino acids, including Thr, can modulate the intestinal microbiota, promoting the multiplication of commensal microorganisms and that the mechanisms involved in this modulation are likely related to mucin synthesis. Mucin production is closely related to dietary Thr levels, and high dietary Thr levels help protect the intestinal epithelium [5]. Furthermore, mucins are rich in Thr and limited dietary Thr may result in decreased mucin biosynthesis, affecting the mucosal protective layer [4]. Similar to previous results reported with dietary supplementation with Thr, supplementation *in ovo* in the present study resulted in greater *MUC2* expression, which may have contributed to the establishing commensal microbiota and increased protection against *Salmonella* infection. Higher *MUC2* expression after *in ovo* Thr supplementation was previously reported in chicks [6] as well as in quails [7].

Infectious processes on the intestinal mucosal surface may result in rapidly releasing of stored mucin granules, thus strengthening the protection provided by the intestinal barrier [28]. Mucin is the main component of the protective mucosal layer and acts as a protective layer of the intestinal epithelium, preventing damage and infection by pathogenic bacteria, and as a fixation substrate for commensal bacteria [29]. In addition, the mucosal layer supplies nutrients for establishing commensal microbiota, strengthening intestinal protection through competitive exclusion [30]. The mucin layer supplies an adequate environment for brush border enzymes to function, thus aiding nutrient absorption [25].

This rationale supports our findings, where *in ovo* Thr supplementation resulted in increased mucin (*MUC2*) and immunoglobulin A (*IgA*) expression, which may have conferred higher protection to animals from the T-SE group. Kadam et al. [11] observed that *in ovo* Thr supplementation improved the humoral immune response, indicating that Thr supplementation resulted in increased immunoglobulin synthesis.

In the present study, broiler chicks from the T-SE group showed similar performance compared to animals from T-SHAM and NT-SHAM. These results might be associated with preserved intestinal mucosal integrity since the T-SE animals presented similar morphometry to unchallenged animals (T-SHAM and NT-SHAM), while chicks from nonsupplemented groups presented compromised mucosal integrity at all times evaluated (24, 96, and 168 hpi). Improved intestinal integrity was also observed in *Salmonella*-challenged chicks fed Thr in the diet [24]. These results indicate that *in ovo* Thr supplementation reduced the burden of *Salmonella* toxins in the gut of broiler chicks. Those toxins have been reported to affect mucin and antimicrobial molecule production and cause cell lysis, apoptosis, rupture of cell junctions, and triggering of inflammatory signaling [4].

## 5. Conclusions

*In ovo* Thr supplementation ameliorated the detrimental effects of *Salmonella* Enteritidis infection, decreased bacterial counts in the cecal content (168 hpi or 9 days of age), and preserved intestinal mucosal integrity, resulting in higher final weight and weight gain. The processes through which *in ovo* Thr supplementation improves broiler response to being challenged with *Salmonella* likely involve increased intestinal mucin synthesis and mucosal antibodies (IgA) and faster maturation of the gastrointestinal tract during embryogenesis.

## Data Availability

The data used to support the findings of this study are included within the article.

## Supplementary Materials

Supplementary Materials Supplementary Table 1: villus height (VH), crypt depth (CD), villus : crypt ratio (V : C), and villus area (area) of broilers at hatch and at two days of age following *in ovo* feeding with threonine. Supplementary Table 2: villus height (VH), crypt depth (CD), villus : crypt ratio (V : C), and villus area (area) of broilers supplemented with threonine *in ovo*, 24-hour postinoculation with *Salmonella* Enteritidis. Supplementary Table 3: villus height (VH), crypt depth (CD), villus : crypt ratio (V : C), and villus area (area) of broilers supplemented with threonine *in ovo*, 96-hour postinoculation with *Salmonella* Enteritidis. Supplementary Table 4: villus height (VH), crypt depth (CD), villus : crypt ratio (V : C), and villus area (area) of broilers supplemented with threonine *in ovo*, 168-hour postinoculation with *Salmonella* Enteritidis.
