# Supplementary material for: Intra-Amnionic Threonine Administered to Chicken Embryos Reduces Salmonella Enteritidis Cecal Counts and Improves Posthatch Intestinal Development
Source: J Immunol Res. 2018 Nov 26;2018:9795829. doi: 10.1155/2018/9795829 (PMC6287158; doi:10.1155/2018/9795829)
Supplement: Supplementary Materials — Supplementary Table 1: villus height (VH), crypt depth (CD), villus : crypt ratio (V : C), and villus area (area) of broilers at hatch and at two days of age following in ovo feeding with threonine. Supplementary Table 2: villus height (VH), crypt depth (CD), villus : crypt ratio (V : C), and villus area (area) of broilers supplemented with threonine in ovo, 24-hour postinoculation with Salmonella Enteritidis. Supplementary Table 3: villus height (VH), crypt depth (CD), villus : crypt ratio (V : C), and villus area (area) of broilers supplemented with threonine in ovo, 96-hour postinoculation with Salmonella Enteritidis. Supplementary Table 4: villus height (VH), crypt depth (CD), villus : crypt ratio (V : C), and villus area (area) of broilers supplemented with threonine in ovo, 168-hour postinoculation with Salmonella Enteritidis. [file 9795829.f1.pdf]

Supplementary Table 1. Villus height (VH), crypt depth (CD), villus:crypt ratio (V:C) and villus area (Area) of broilers at hatch and at two days of age following *in ovo* feeding with Thr.

	Age			
Duodenum	Hatch		Day 2	
	NT	T	NT	T
VH (µm)	475.32 b	492.03 a	869.86 b	977.90 a
CD (µm)	30.65 a	27.27 b	50.56 a	46.89 b
V:C (µm/µm)	15.55 b	18.19 a	17.31 b	20.84 a
Area (mm^2)	0.123 b	0.143 a	0.355 b	0.462 a
Jejunum	Hatching		Day 2	
	Control	Thr	Control	Thr
VH (µm)	315.03 a	311.22 a	501.68 b	549.12 a
CD (µm)	25.00 a	20.86 b	44.96 a	41.78 b
V:C (µm/µm)	12.76 b	14.98 a	12.08 b	13.96 a
Area (mm^2)	0.064 a	0.065 a	0.186 a	0.190 a

Means followed by the same letter within the same column did not significantly differ by the F test at $p \leq 0.05$.

Supplementary Table 2. Villus height (VH), crypt depth (CD), villus:crypt ratio (V:C) and villus area (Area) of broilers supplemented with Thr *in ovo,* 24 hours post-inoculated with *Salmonella* Enteritidis (hpi).

		Duodenum		
Treatment	VH (µm)	CD (µm)	V:C (µm/µm)	Area (mm^2)
NT-SHAM	1210.18 b	61.92 b	19.57 b	0.574 b
NT-SE	1045.81 c	72.95 a	14.41 d	0.462 c
T-SHAM	1325.76 a	57.24 c	23.28 a	0.702 a
T-SE	1181.14 b	63.33 b	18.75 c	0.608 b
		Jejunum		
NT-SHAM	577.58 b	52.89 b	11.01 a	0.215 b
NT-SE	541.81 c	60.86 a	9.15 c	0.203 b
T-SHAM	624.34 a	57.21 ab	10.93 ab	0.267 a
T-SE	620.18 a	60.33 a	10.41 b	0.251 a

Means followed by the same letter within the same column and tissue did not significantly differ by Tukey's test at $p \leq 0.05$.NT-SHAM: no *in ovo* Thr supplementation and sham-inoculated in the post-hatch challenge; T-SHAM: *in ovo* Thr supplementation and sham-inoculated; NT-SE: no *in ovo* Thr supplementation and SE Nal^R-challenged; and T-SE: *in ovo* Thr supplementation and SE Nal^R-challenged.

Supplementary Table 3. Villus height (VH), crypt depth (CD), villus:crypt ratio (V:C) and villus area (Area) of broilers supplemented with Thr *in ovo,* 96 hours post-inoculated with *Salmonella* Enteritidis (hpi).

Treatment	VH (μm)	CD (μm)	V:C (μm/μm)	Area (mm^2)
		Duodenum		
NT-SHAM	1423.01 a	56.75 c	25.58 a	0.739 a
NT-SE	1274.78 b	72.62 b	17.78 d	0.625 b
T-SHAM	1440.31 a	69.47 b	21.82 b	0.741 a
T-SE	1338.33 a	84.36 a	20.18 c	0.675 ab
		Jejunum		
NT-SHAM	840.36 a	56.96 b	14.55 b	0.392 ab
NT-SE	816.92 b	62.52 a	13.53 c	0.363 b
T-SHAM	864.03 a	52.24 b	16.44 a	0.430 a
T-SE	822.87 ab	54.92 b	14.94 b	0.382 ab

Means followed by the same letter within the same column and tissue did not significantly differ by Tukey's test at $p \leq 0.05$. NT-SHAM: no *in ovo* Thr supplementation and sham-inoculated in the post-hatch challenge; T-SHAM: *in ovo* Thr supplementation and sham-inoculated; NT-SE: no *in ovo* Thr supplementation and SE Nal^R-challenged; and T-SE: *in ovo* Thr supplementation and SE Nal^R-challenged.

Supplementary Table 4. Villus height (VH), crypt depth (CD), villus:crypt ratio (V:C) and villus area (Area) of broilers supplemented with Thr *in ovo,* 168 hours post-inoculated with *Salmonella* Enteritidis (dpi).

	Duodenum			
Treatment	VH (µm)	CD (µm)	V:C (µm/µm)	Area (mm^2)
NT-SHAM	1600.14 a	94.26 a	16.48 b	0.999 a
NT-SE	1442.26 b	98.54 a	15.73 b	0.755 b
T-SHAM	1622.46 a	82.87 b	19.01 a	1.094 a
T-SE	1556.54 a	86.20 b	18.94 a	1.042 a
	Jejunum			
NT-SHAM	995.85 a	80.46 a	12.02 bc	0.549 b
NT-SE	873.76 b	82.98 a	11.02 c	0.464 c
T-SHAM	975.41 a	71.38 b	14.11 a	0.675 a
T-SE	882.89 b	69.08 b	13.19 ab	0.529 bc

Means followed by the same letter within the same column and tissue did not significantly differ by Tukey's test at $p \leq 0.05$. NT-SHAM: no *in ovo* Thr supplementation and sham-inoculated in the post-hatch challenge; T-SHAM: *in ovo* Thr supplementation and sham-inoculated; NT-SE: no *in ovo* Thr supplementation and SE Nal^R-challenged; and T-SE: *in ovo* Thr supplementation and SE Nal^R-challenged.
